# Fertility issues in women of childbearing age with spondyloarthritis

**DOI:** 10.3389/fimmu.2024.1412174

**Published:** 2024-06-14

**Authors:** Sara Bindoli, Giacomo Cozzi, Mariagrazia Lorenzin, Paolo Sfriso, Andrea Doria, Laura Scagnellato, Roberta Ramonda

**Affiliations:** Rheumatology Unit, Department of Medicine, University of Padova, Padova, Italy

**Keywords:** fertility, spondyloarthritis, pregnancy outcome, bDMARD, hormones, psychological distress

## Abstract

The topic of fertility in women with spondyloarthritis (SpA) has been scarcely investigated to date. Recent systematic reviews and registry studies have brought renewed attention to the plight of women of childbearing age with rheumatic diseases, in particular SpA. Fertility may be impacted by physical impairment, hormonal imbalances and psychological distress. Several studies observed a reduction in anti-Müllerian hormone in women with SpA, reflecting a reduced ovarian reserve (OR). Furthermore, disease activity and the use of certain therapies can alter fertility, and this is reflected in a prolonged time-to-pregnancy (TTP), a validated outcome measure that can evaluate the status of subfertility. The employment of glucocorticoids or non-steroidal anti-inflammatory drugs has also been linked to reduced fertility, whereas the use of biologics, especially tumour necrosis factor inhibitors (TNFi), is not associated with a prolonged TTP. In all women of childbearing age with rheumatic diseases, preconception counselling is paramount, and a referral to a reproductive specialist should be considered in the presence of multiple factors that may influence fertility. A comprehensive evaluation involving a multidisciplinary team of rheumatologists, gynaecologists, and often psychologists is warranted. In this narrative review, we collected the currently available literature focusing on fertility issues in women affected by SpA, providing data on fertility outcomes, hormonal imbalance, and therapeutic concerns.

## 1 Introduction

**Figure 1:**
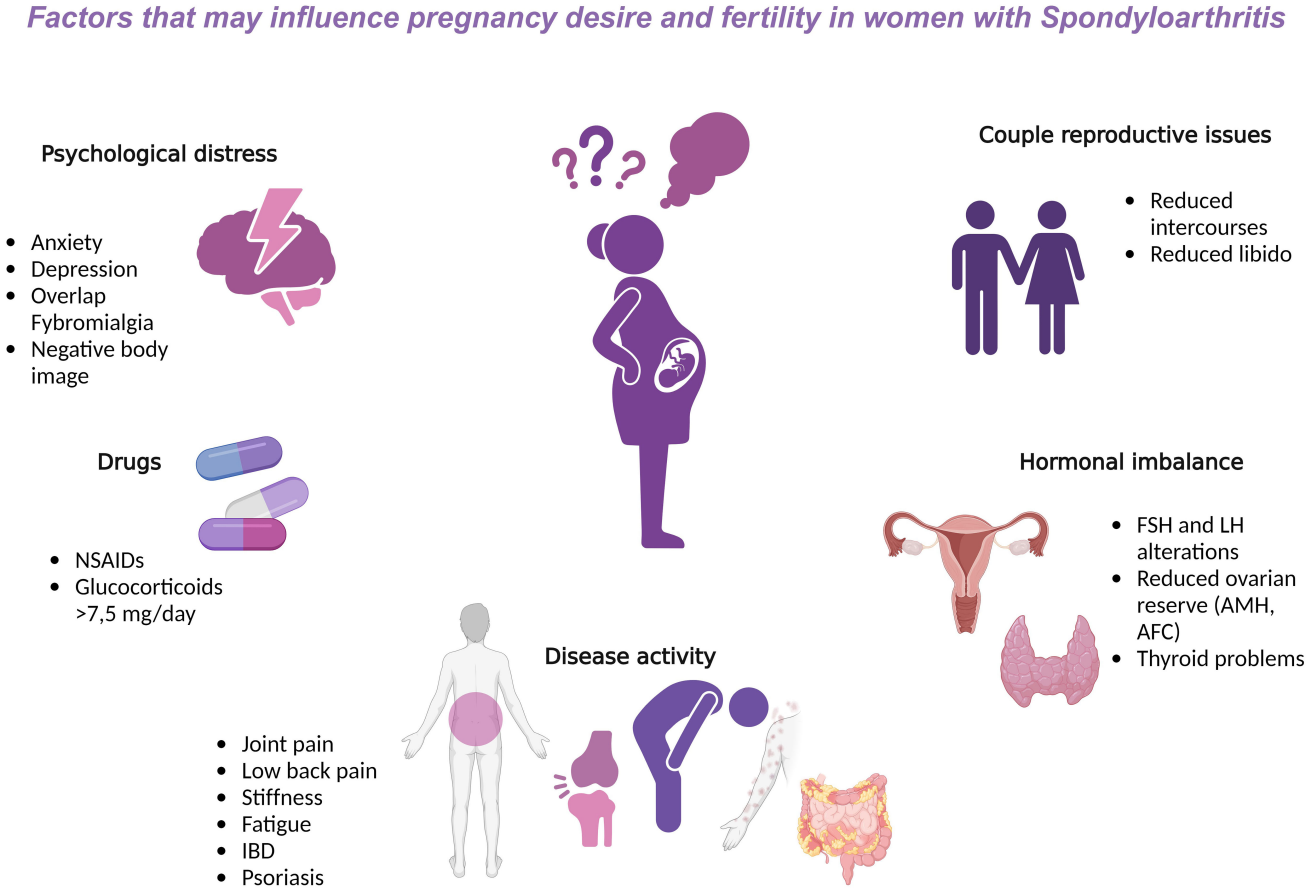
Factors that may influence pregnancy desire and fertility in women with Spondyloarthritis. AFC, antral follicle count; AMH, anti-Müllerian hormone; b, biological; csDMARDs, conventional synthetic Disease modifying antirheumatic drugs; FSH, follicle stimulating hormone; IBD, intestinal bowel disease; LH, luteinizing hormone; NSAIDs, non-steroidal anti-inflammatory drugs; TNF, Tumor Necrosis Factor.

The topic of fertility in rheumatological patients has been widely discussed in recent years, and reproductive issues in patients with chronic diseases are becoming more known. Rheumatic diseases (RD) may affect the quality of life and reproduction in both sexes (1, 2). Fertility issues in women with RD may occur in diseases with extensive systemic inflammation and autoantibody production as well as all joint diseases (3). RD can have a great impact on quality of life, causing disability, stiffness, and pain, all factors that greatly interfere with the reproductive and then the psychological health of those affected; indeed, anxiety, stress, depression, or negative body image, can negatively impact the quality of reproductive health. In addition, therapies and treatments employed can hamper the maternity desire, and hormonal imbalances, not infrequent in childbearing women, may aggravate the problem. Altogether, these factors are finally responsible for a reduced frequency of intercourses, loss of libido and general sexual satisfaction with the partner (4) (**Figure 1**).

The possible fertility reduction in women with RD has been reported in several studies, particularly those including patients with rheumatoid arthritis (RA) and systemic lupus erythematosus (SLE) (5), but there only a few retrospective studies, mainly derived from registries, and two systematic reviews (6, 7) specifically focused on fertility/pregnancy outcomes in spondyloarthritis (SpA), to date. A questionnaire-based study involving a representative sample of rheumatologists and obstetricians observed that most physicians perceive SpA as the “safest” RD in terms of fertility and pregnancy outcomes, compared to RA, SLE, systemic sclerosis, or vasculitis (8).

**Figure 2:**
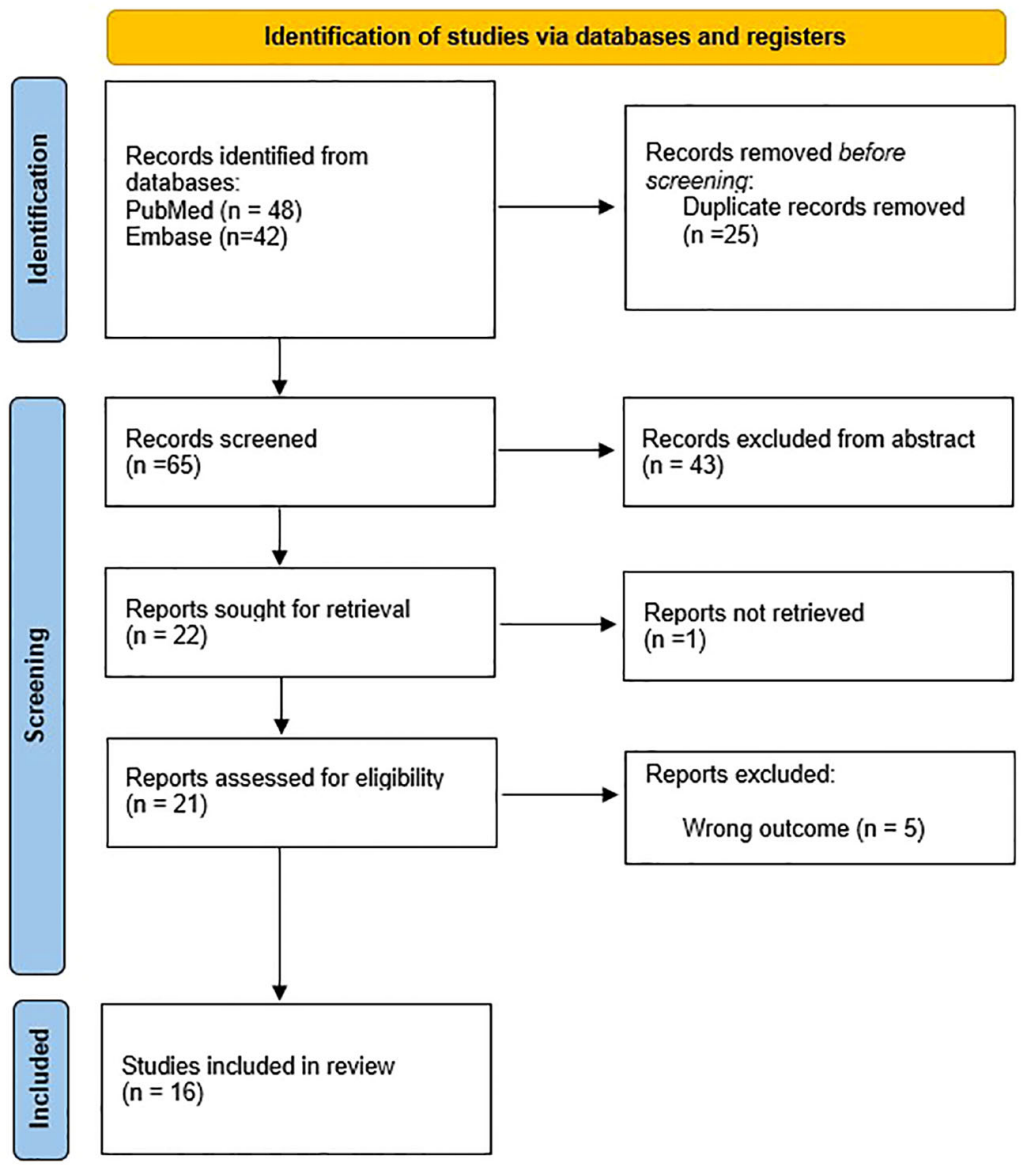
PRISMA flowchart.

We endeavoured to conduct a narrative review with an online literature search on Medline via PubMed and on Embase via Ovid. The search strategy included all MeSH/Emtree synonyms and free terms of “spondyloarthritis”, along with the term “fertility”. The selection strategy, done using the Rayyan software (9), is depicted in the PRISMA (10) flowchart (**Figure 2**).

## 2 Axial spondyloarthritis: sex-related differences

Spondyloarthritis is the second most prevalent chronic RD in both sexes and in particular, women of childbearing age (11). Axial (ax)SpA can affect the spine, as well as entheses and joints, causing structural damage, including new bone formation in the axial and peripheral skeleton. The disease may presents both in a radiographic form (r-axSpA) with structural changes of the sacroiliac (SI) joint, according to the modified New York criteria (12), and a non-radiographic form (nr-axSpA), characterised by the absence of x-ray findings on the spine, entheses and joints. The worldwide prevalence is about 1% and the disease with the usual disease onset <45 years old (13). Although the male-female prevalence ratio has historically shown a large overestimation favouring males, a steady decline has been reported (14). Unlike r-axSpA, there is little difference in the prevalence of nr-axSpA in men vs. women (15–18). It bears reminding that males and females have different immunological profiles as the former are more prone to high levels of interleukin (IL)-17A and IL-18, whereas the latter express more IL-6. Additionally, hormones might also play a role, despite conflicting evidence across different studies (19–22). Studies have revealed that ineffective treatment with TNFi results in significantly higher Bath Ankylosing Spondylitis Disease Activity (BASDAI) and lower quality of life (QoL) scores in women vs. men (14). Therefore, although men with axSpA usually have a worse radiographic prognosis, women have a higher disease burden, mostly due to the aforementioned factors (14).

## 3 Risk factors of subfertility in SpA women of childbearing age

### 3.1 Hormones

Fertility is the capacity to achieve pregnancy, whereas fecundity is the capacity to have a live birth, which includes gamete production, fertilisation, and the ability to carry a pregnancy [2]. Subfertility is the failure to establish a clinical pregnancy after 12 months of regular and unprotected sexual intercourse and it is measured using a parameter called time-to-pregnancy (TTP) (23). Fertility rate is defined as the average number of children per woman in her lifetime [2]. Pregnancy outcomes, TTP and personal choices may influence fertility rate. A decreased fertility rate in women with rheumatoid arthritis (RA) has long been described (24), but there is scant data about fertility in women with SpA. Fertility may be assessed by the ovarian reserve (OR) (25), which can be determined by the antral follicle count (AFC), serum anti-Müllerian hormone (AMH) and basal follicle-stimulating hormone (FSH) levels (26). Serum AMH levels, secreted by granulosa cells during fertile years, has a high predictive value for fertility potential in a subfertile population (27), as it reflects the remaining follicle pool and may therefore be used as a marker of OR. Recent studies reported reduced levels of AMH or AFC in patients with SLE, Takayasu arteritis, Behçet’s disease (BD) and primary antiphospholipid syndrome (28–31), whereas other studies were unable to demonstrate a reduction in OR in patients with RA or Crohn’s disease (32, 33). In a study by Henes et al., women with RA, SpA and BD had overall lower AMH levels vs. healthy women (34). In particular, the presence of human leukocyte antigen (HLA)-B27 was associated with significantly reduced AMH levels in SpA, differently from RA wherein the presence of rheumatoid factor (RF) and anti-citrullinated protein antibodies (ACPA) did not correlate with reduced OR (34). These findings were corroborated in a study by Brouwer et al., in which AMH levels in women with newly diagnosed RA who had not received pharmacotherapy were not significantly different from age-matched controls, either at diagnosis or six months after the disease onset (33). Similarly, an observational study found that AMH levels were lower in a cohort of premenopausal women with arthritis vs. healthy controls, and patients with accelerated reduction in AMH levels were more likely >35 years old (35). Recently, a Turkish study observed a reduced OR reflected by lower AMH, AFC and baseline FSH levels in women with SpA compared to controls (36). Although the data appears to confirm an overall decrease in OR in SpA women, the use of biological disease-modifying antirheumatic drugs (bDMARDs) does not greatly impair fertility. A recent report supported the use of bDMARDs in SpA women of childbearing age, given that AMH levels were not influenced by the treatment or disease activity (37). Moreover, studies have reported that fertilisation might be difficult if pro-inflammatory cytokines, particularly TNF-α, are overexpressed; therefore, the use of TNFi could improve and sustain the fertility rate in SpA (38). Overall, bDMARDs are paramount to maintain the disease under control, and avoid flares as well as the worsening of the proinflammatory state, which can enhance both pain and psychological distress.

### 3.2 Use of pre-conceptional non-steroidal anti-inflammatory drugs and steroids

Studies focused on fertility rate in real-life cohorts of women with SpA are very scarce and most data derives from one of the largest multicentre registries (the Norwegian nationwide registry “RevNatus”). The study aimed to assess TTP, and factors associated with prolonged TTP in women with axSpA and RA (39). Data from 274 women with axSpA and 317 with RA were collected. The fertility outcome was as follows: 93.8% of the women became pregnant with a median TTP of 2 months, whereas the 21.2% had TTP>12 months. Overall, a median TTP of 4 months was reported in those who were planning a pregnancy at the time of enrolment, with approximately 32% having a TTP>12 months. In addition, 7.7% of the women underwent fertility treatment. Factors that were most associated with a prolonged TTP were older age, nulliparity and longer disease duration. However, there was no prolonged TTP according to disease activity or medication, including the preconception use of non-steroidal anti-inflammatory drugs (NSAIDs), prednisolone, methotrexate (MTX), or TNFi, in both fertile and sub-fertile women with axSpA, in contrast to previous studies that reported a prolonged TTP when NSAIDs or glucocorticoids >7.5 mg/day are employed (4). No differences in health-related quality of life (HrQoL) were observed in sub-fertile vs. fertile axSpA, even though pain was considerable in all the subgroups; BASDAI or HRQoL and BMI were not factors significantly associated with a prolonged TTP, despite previous reports suggesting that a higher Body Mass Index (BMI) may affect fertility (40). A recent French multicentre study (NCT02450396) comprising 88 women of childbearing age with SpA, reported a median TTP of 16.1 months in 56 subjects, with a subfertility rate of 45%. The use of pre-conceptional NSAIDs, was associated with an even more prolonged TTP 31.6 months [95% CI (22.3–40.4)] vs. 12.3 months [95% CI (10.9–20.3)] in women with no exposure to NSAIDs (p=0.01). Moreover, this study also confirmed older age as an independent factor for prolonged TTP (41).

### 3.3 Age, nulliparity and disease activity

In the comparison between RA and SpA women, subfertility was found to be more common in the former. The “PARA study” assessed the TTP in RA women and found a TTP >12 months in 42% of the 245 patients analysed (42), a higher proportion than that observed in the Norwegian cohort of SpA (39). The factors related to a prolonged TTP were older age, nulliparity, high disease activity (measured through DAS-28), and the pre-conceptional use of NSAIDs and prednisone >7.5 mg/day. Indeed, according to this study, high-dose users of prednisone had a longer TTP vs. low-dose users (p=0.04) and vs. patients not receiving prednisone (p=0.002). However, the TTP between the latter two did not differ significantly (42). Other factors that may be associated with a prolonged TTP include the presence of past gynaecological conditions, the infertility of the partner and the psychological burden of active SpA. Thus, before planning a pregnancy it is of the utmost importance to ensure that the disease is under control, possibly avoiding the use of NSAIDs and glucocorticoids at the preconception stage.

### 3.4 The psychological burden

A global assessment on fertility and sexuality in women affected by RA, SpA, psoriatic arthritis (PsA), and juvenile chronic arthritis diagnosed before 40 years old and between 18–50 years old was conducted in a retrospective questionnaire-based study carried out in France in 202 subjects (43). The rate of spontaneous pregnancies and TTP were the relevant outcomes evaluated. Before receiving the diagnosis, the rate of spontaneous pregnancies was found to be similar in RA, PsA, and SpA. Conversely, post-diagnosis, the rate of spontaneous pregnancies showed a significant decrease in patients with RA, though not statistically significant in SpA and PsA. However, TTP was not significantly different between the three groups. Overall, 81% of patients (all types of inflammatory arthritis combined) achieved spontaneous pregnancy, compared to 96% before diagnosis, and there was no significant difference between the groups. Furthermore, two-thirds of patients reported a decrease in the frequency of sexual intercourse after diagnosis, mainly due to the pain related to the disease or low libido, and only a minority of women (9%) consulted a specialist regarding the absence of spontaneous pregnancy. The impact on the psychological health and pain, reflected by diminished sexual activity, should be considered as a relevant aspect that should be managed globally by the rheumatologists, psychologists and gynaecologists, hence the need for a multidisciplinary approach.

### 3.5 Other possible causes of subfertility

Two reviews were carried out to ascertain the impact of SpA and related therapies on fertility, pregnancy and disease activity during pregnancy (6, 7). A first systematic literature review conducted in 2017, did not find any links between SpA and infertility, or between SpA and pregnancy, delivery or foetal complications (7). A 2022 systematic review included 21 studies, comprising a total of 3718 pregnancies in 3566 patients (2848 with axSpA and 718 with PsA) (6). Regarding disease activity, 9 out of 12 studies on axSpA found no changes in disease activity during pregnancy, with only one study observing some improvement (44) and two a worsening (45, 46). As regards fertility, only 4 studies on SpA were evaluated: 2 comprising 991 patients with axSpA (47, 48), and 2 comprising 114 patients with PsA (49, 50). Both retrospective studies on axSpA found no evidence of self-reported fertility impairment. The case-control study on PsA yielded no significant findings. However, the study by Eudy et al., found a self-reported infertility rate of 36%, primarily due to polycystic ovary syndrome (50% of cases) (49). Another recently identified cause of infertility in SpA is endometriosis, though the underlying mechanisms linking endometriosis to SpA remain unclear, some studies have suggested that they may share the same genetic basis (51).

## 4 Pregnancy outcomes

Pregnancy outcomes were also evaluated in 9 studies comprising a total of 2527 axSpA women, and 5 studies comprising 573 PsA patients. Overall, there was a significant increase in prematurity for both axSpA and PsA; similarly, an increase in Caesarean sections was also observed for both diseases, with a specific over-risk for elective Caesarean section in PsA. In axSpA, there was also a 2-fold increased risk of small-for-gestational-age (SGA) and an increased risk of preeclampsia (6). Similar findings were later confirmed in a study using data derived from the Ankylosing Spondylitis Registry of Ireland (ASRI); the Authors found an increase in preterm births, preeclampsia and low birth weight in axSpA women, whereas SGA was less prevalent vs. the general population (52).

## 5 SpA disease activity

There is evidence that pregnancy has no or mild effects on disease activity in SpA, however, patients usually report an increase in pain unlike in RA (53). Indeed, previous studies observed that in pregnant women with SpA, the levels of T-regulatory (Treg) cells, expressed by FOXP3, were lower in comparison to healthy pregnant women. Therefore, it was hypothesized that Treg cells of pregnant women with SpA are unable to foster an anti-inflammatory cytokine environment, thereby leading to a persistent inflammatory state (54).

Data from the ASRI registry described no changes or a stable disease before conception in 72.4% of the 98 women included. However, an increase in disease activity scores was reported during pregnancy in 31.6% of them, with less than 20% reporting an improvement in disease activity during this time (52). An unchanged or slightly worsened disease was observed in the cohort of Eudy et al., for axSpA, whereas an unchanged or milder disease was recorded in PsA women (49). Finally, a Turkish study observed a 23–24.5% rate of flares during pregnancy in SpA, a finding comparable with RA patients, but lower vs. vasculitis or SLE. In contrast, postpartum flares in SpA remained low vs. RA, SLE, and vasculitis (8).

## 6 Anti-rheumatic therapies and fertility

Antirheumatic drugs may affect fertility by hampering ovulation as well as interfering with pituitary and gonadal hormone production. Women with RD may experience subfertility due to medications that increase the risk of miscarriage. Several drug classes are employed in SpA, such as NSAIDs, glucocorticoids, conventional synthetic (cs)DMARDs, as well as bDMARDs. Non-selective and selective cyclooxygenase inhibitors may induce luteinised unruptured follicle syndrome in a dose-dependent and menstrual cycle-dependent manner (55). Sulfasalazine is not associated with reduced fertility (56), and therefore there is no restriction on its use nor is it teratogenic. Glucocorticoids are known to provoke a prolonged TTP, especially with doses >7.5 mg (33), by interfering with the pituitary-gonadal axis, with impaired secretion of FSH and luteinising hormone (LH). In addition, glucocorticoids may directly bind the glucocorticoid receptor on ovarian cells (57). Methotrexate is a teratogenic drug that must be avoided during pregnancy, but no effects on OR have been observed (33). Among bDMARDs, TNFi are commonly employed to treat SpA and there is no evidence of altered fertility associated with TNFi in both males and females with SpA (58). In fact, TNFi may improve fertilisation by promoting an anti-inflammatory environment, and therefore their use should not be precluded in SpA women of childbearing age. Nevertheless, there is insufficient data, except for certolizumab and etanercept, which can be continued throughout pregnancy (59), to establish the safety of TNFi — as well as IL-17, IL-23 and JAK inhibitors — throughout pregnancy and breastfeeding (4, 60).

## 7 Family planning and counselling

In women with RD of childbearing age, it is important to assess and discuss desire for pregnancy desire during routine follow-up visits. It is paramount to ascertain pre-conceptional risk and offer pregnancy counselling in women with systemic autoimmune and autoinflammatory diseases. Several factors should be considered, such as the age of the patient, the outcome of previous pregnancies if any, pain levels, disease activity or occurrence of recent flares, and current treatments — with particular attention to any drug with pregnancy contraindications (61). Once the patient formulates their desire for pregnancy, a dedicated consult is necessary to ensure disease remission and educate on the impact of disease activity on fertility and pregnancy outcomes. It is advised to perform vitamin deficiency tests, ascertain vaccination status, and decide on a management plan for both preconception and pregnancy. Moreover, it is important that patients undergo gynaecological evaluation for general risk factors (e.g. obesity, hypertension, diabetes, etc.), and the presence of severe spine involvement with hypomobility of the lumbar area should prompt specific counselling about modes of delivery (62). The main limitation of pre-pregnancy counselling is that not all physicians are comfortable talking about sexual activity with RD women, likely due to a lack of specific skills or deep knowledge of fertility and pregnancy outcomes associated with the specific disease. Furthermore, counselling may be time-consuming and not all the referral Centres are equipped to deal with such complex patients with expressed desire for pregnancy. It is important to achieve sustained minimal disease activity, and to avoid flares to create suitable conditions for a pregnancy. Finally, a referral to a reproduction specialist should be considered in the presence of additional pregnancy obstacles, such as psychological distress and other conditions unrelated to the patient (e.g. the partner’s fertility issues). Thus, there is a need for a multidisciplinary evaluation of the patient who desires pregnancy including rheumatologists, gynaecologists, as well as psychologists.

Although there is scant data on fertility in SpA, early family planning and pre-conceptional screenings are crucial to improve patients’ experience during pregnancy and treatment choices. This strategy is essential to better prepare and ensure a successful pregnancy and help optimise both maternal and neonatal health outcomes.

## Author contributions

SB: Conceptualization, Visualization, Writing – original draft, Writing – review & editing, Investigation. GC: Writing – review & editing, Investigation. ML: Writing – review & editing, Conceptualization. AD: Supervision, Visualization, Writing – review & editing. PS: Supervision, Writing – review & editing. LS: Writing – review & editing, Conceptualization. RR: Conceptualization, Supervision, Visualization, Writing – review & editing, Investigation, Writing – original draft.
